# Correction: A conserved strategy for inducing appendage regeneration in moon jellyfish, *Drosophila*, and mice

**DOI:** 10.7554/eLife.90007

**Published:** 2023-06-22

**Authors:** Michael J Abrams, Fayth Hui Tan, Yutian Li, Ty Basinger, Martin L Heithe, Anish Sarma, Iris T Lee, Zevin J Condiotte, Misha Raffiee, John O Dabiri, David A Gold, Lea Goentoro

**Keywords:** *D. melanogaster*, Mouse

Abrams NJ, Tan FH, Li Y, Basinger T, Heithe ML, Sarma A, Lee IT, Condiotte ZJ, Raffiee M, Dabiri JO, Gold DA, Goentoro L. 2021. A conserved strategy for inducing appendage regeneration in moon jellyfish, *Drosophila*, and mice. *eLife* **10**:e65092. doi: 10.7554/eLife.65092.

Published 7 December 2021

There was an error in the Materials and methods of this article. This error (which was in the fourth sentence of the section "Regeneration experiments") has now been corrected.

Corrected text: Recovering *Drosophila* were allocated randomly to vials with standard lab fly food (control) or standard lab fly food mixed with 1.7 mM L-Leucine (Sigma-Aldrich L8000), 1.7 mM L-Glutamine (Sigma-Aldrich G3126), and 33 μg/ml insulin (human recombinant, MP Biomedicals 0219390080).

Original text: Recovering *Drosophila* were allocated randomly to vials with standard lab fly food (control) or standard lab fly food mixed with 5 mM L-Leucine (Sigma-Aldrich L8000), 5 mM L-Glutamine (Sigma-Aldrich G3126), and 0.1 mg/ml insulin (human recombinant, MP Biomedicals 0219390080).

The article has been corrected accordingly.

