# Supplementary material for: A conserved strategy for inducing appendage regeneration in moon jellyfish, Drosophila, and mice
Source: eLife. 2021 Dec 7;10:e65092. doi: 10.7554/eLife.65092 (PMC8782573; doi:10.7554/eLife.65092)
Supplement: Figure 3—source code 1. [file elife-65092-fig3-code1.zip › Rcodes.rtf]

To run these codes: * Make sure you have the metaphor package. If not download the metafor package* Save the regeneration data tables provided in Aurelia Regeneration Data.xlsx into csv files. The codes are written here with the headings used in the tables provided in the excel file. See additional note below for detailed formatting of the data file.  * To perform statistical analysis on an effect size measure, copy and paste the relevant commands into the R console   * Please see Viecthbauer, 2010 for more detailed description of the metaphor functions escalc and rma, or use the help function in R To compute Risk Ratio (RR) statistics: Upload the metafor package>library(metafor)Specify the function statRR>statRR <- function(filename, model, confidence) {effect <- read.csv(file=filename, head=TRUE, sep=",")effectRR <- escalc(measure="RR", ai=treg, bi=tnoreg, ci=creg, di=cnoreg, data=effect)effectRRstat <- rma(yi,vi,data=effectRR, level=confidence, method=model)print(effectRRstat)predict(effectRRstat,transf=exp,digit=2)}Run the function statRR >statRR("thefilename.csv", model="FE", 95)where the function statRR uploads a data file called the thefilename.csv, and uses the Fixed-Effect (FE) model to compute 95% confidence intervals of the RR measure. See rma function and Viecthbauer, 2010 for description of statistical models used in the metafor package. To compute Odds Ratio (OR) statistics: Upload the metafor package>library(metafor)Specify the function statOR>statOR <- function(filename, model, confidence) {effect <- read.csv(file=filename,head=TRUE,sep=",")effectOR <- escalc(measure="OR",ai=treg, bi=tnoreg, ci=creg, di=cnoreg, data=effect)effectORstat <- rma(yi,vi,data=effectOR,level=confidence,method=model)print(effectORstat)predict(effectORstat,transf=exp,digit=2)}Run the function statOR>statOR("thefilename.csv", model="FE",95)where the function statOR uploads a data file called the thefilename.csv, and uses the Fixed-Effect (FE) model to compute 95% confidence intervals of the OR measure. See rma function and Viecthbauer, 2010 for description of statistical models used in the metafor package. To compute Response Ratio (R) statistics: Upload the metafor package>library(metafor)Specify the function statR>statR <- function(filename, model, confidence) {effect <- read.csv(file=filename, head=TRUE, sep=",")effectR <- escalc(measure="ROM", m1i=m2i, m2i=m1i, sd1i=sd2i, sd2i=sd1i, n1i=n2i, n2i=n1i,  data=effect)effectRstat <- rma(yi,vi, data=effectR, level=confidence, method=model)print(effectRstat)predict(effectRstat,transf=exp,digit=2)}Run the function statR statR("thefilename.csv", model="FE",95) where the function statR uploads a data file called the thefilename.csv, and uses the Fixed-Effect (FE) model to compute 95% confidence intervals of the R measure. See rma function and Viecthbauer, 2010 for description of statistical models used in the metafor package. Formatting of datafile Tables of regeneration data can be copy-and-pasted from Aurelia Regeneration Data.xlsxCopy and paste the regeneration table into a new Excel file, and then save the file as a csv fileIf you get an error message, there could be an extra line automatically created in the csv file. If so remove the extra line. The functions statRR and statOR call a csv data file of a 2 x 2 table of data. For example: ExpID,creg,cnoreg,treg,tnoreg58,56,30,8,3461,36,53,26,5668,17,72,14,7078,25,55,2,74where each row is a result from an independent biological replicate (indicated by the Exp ID), and the columns are: creg 		= number of ephyrae that regenerate in controlcnoreg	= number of ephyrae that do not regenerate in controltreg 		= number of ephyrae that regenerate in treatmenttnoreg	= number of ephyrae that do not regenerate in treatmentThe functions statR call a csv data file of a 2 x 2 table of data. For example: Exp ID,m1i,m2i,sd1i,sd2i,n1i,n2i58,0.41102678,0.461509069,0.195857086,0.204522107,6,3761,0.294927983,0.318230799,0.128414053,0.139916644,49,7368,0.430886755,0.400797935,0.235476494,0.182167134,33,3478,0.243601412,0.417921024,0.078973556,0.224103527,20,3279,0.25602563,0.37092022,0.091076218,0.157526878,29,97where each row is a result from an independent biological replicate (indicated by the ExpID), and the columns are: m1i 		= mean measurement (of e.g., arm length) in controlm2i		= mean measurement in treatmentsd1i 		= standard deviation of the measurement in controlsd2i		= standard deviation of the measurement in treatmentn1i		= sample size in controln2i		= sample size in treatment
